# A Novel Multi-Criteria Decision-Making Model for Building Material Supplier Selection Based on Entropy-AHP Weighted TOPSIS

**DOI:** 10.3390/e22020259

**Published:** 2020-02-24

**Authors:** Chun-Ho Chen

**Affiliations:** Professional Architect and Urban Planner, Bachelor Program of Real Estate Investment and Management, Takming University of Science and Technology, No.56, Sec. 1, Huanshan Rd., Neihu District, Taipei 11451, Taiwan; phdchen5598@takming.edu.tw

**Keywords:** decision-making model, entropy-AHP weight, combination weighting method, TOPSIS

## Abstract

The type of criterion weight can be distinguished according to different decision methods. Subjective weights are given by decision makers based on their knowledge, experience, expertise, and other factors. Objective weights are obtained through multi-step calculations of the evaluation matrix constructed from the actual information about the evaluation criteria of the alternatives. A single consideration of these two types of weights often results in biased results. In addition, in order to build an effective supply chain source, buyers must find suitable quality products and/or service providers in the process of supplier selection. Based on the above reasons, it is difficult to accurately select the appropriate alternative. The main contribution of this paper is to combine entropy weight, analytic hierarchy process (AHP) weight, and the technique for order preference by similarity to an ideal solution (TOPSIS) method into a suitable multi-criteria decision making (MCDM) solution. The TOPSIS method is extended with entropy-AHP weights, and entropy-AHP weights are used instead of subjective weights. A novel decision-making model of TOPSIS integrated entropy-AHP weights is proposed to select the appropriate supplier. Finally, we take the selection of building material suppliers as an example and use sensitivity analysis to show that the combination of the TOPSIS method based on entropy-AHP weights can effectively select the appropriate supplier.

## 1. Introduction

The decision-making process usually needs to consider multiple criteria at the same time, and requires multiple standard technologies to assist decision making. In the field of multi-criteria decision-making conditions, decision makers should follow the principle of rationality when choosing the most suitable alternative, that is, to assess a limited set of interdependent or independent criteria [1,2].

Supply chain management (SCM) is mainly to improve competitiveness, optimize business processes, cycle and inventory levels, maximize revenue and profitability, reduce manufacturing costs, improve customer satisfaction, and meet consumer services [3,4,5,6,7,8]. In the decision-making method of supply chain management, especially in some complex areas, certain criteria need to be defined in advance. In the early stages of the supply chain, choosing the most favorable supplier is one of the most important tasks [9]. Academia and practice have focused extensively on supply chain management in recent years.

The effective procurement mechanism is one of the necessary conditions for a successful supply chain [3,10,11,12]. The right supplier selection can save the company huge costs and is also an important responsibility of the purchasing agency [13]. Several methods of supplier suitability selection are proposed for discussion. Supplier selection in systematic analysis, including the analytic hierarchy process (AHP) [14,15], the supplier performance matrix approach [16], vendor profile analysis [17], the matrix approach [18], the weighted point method, and taxonomy [19,20].

The supplier selection problem is diversified and contains the characteristics of multi-indicator standards, complexity, and non-structure. This is a multi-standard selection problem [21,22,23,24].

Decision-making tasks often ignore subjective and objective factors, such as the failure to consider data information, an incorrect expression of preferences, qualitative criteria, and other qualitative criteria [25,26]. Most methods are designed to solve the problem of supplier selection in non-complex situations [27]. The method of the fuzzy analytic hierarchy process was proposed and constructed as a structural model for rubber supplier evaluation [13]. 

Hwang and Yoon proposed a well-known and commonly used multi-criteria decision making (MCDM) method, called TOPSIS, which is the technique for order preference by similarity to an ideal solution [28]. The TOPSIS method includes both a positive ideal solution of the benefit type and a negative ideal solution of the cost type. A suitable alternative should be closer to positive ideal solutions, but away from negative ideal solutions.

The academic community attaches great importance to TOPSIS’s research method and regards it as one of the main research topics, because it can solve supplier selection objectively and effectively [29,30]. However, the weight of TOPSIS must be determined subjectively by policy makers, so its shortcomings still exist [2,31]. In the final stage of the analysis, we rank all alternatives with TOPSIS and select the most suitable alternative [32].

To effectively solve supplier selection issues, a novel decision-making model of TOPSIS integrated entropy-AHP weight is proposed to select the appropriate supplier. The following in-depth analysis is based on the above information, and the main research themes of this paper are as below:

(1) TOPSIS has its shortcomings in setting weights, and its weight is subjectively judged by decision makers. Therefore, when the information obtained by decision makers is incomplete or the subjective consciousness of the decision makers is too strong, how should such errors be resolved? 

(2) The measurement of the first-layer entropy weight is different from the second-layer entropy weight. How do we combine the TOPSIS method with entropy to obtain objective weight values? 

(3) The weight obtained by the entropy weight method is an objective weight, which can make up for the deficiency of the subjective weight obtained by AHP. How should we obtain the entropy-AHP weight based on the two weights of the entropy weight method and the AHP method, and apply it to the TOPSIS method?

In this study, in order to choose a suitable building material supplier, we propose a novel decision-making model of the TOPSIS integrated entropy-AHP weight. The entropy-AHP weighted TOPSIS method has huge application and success opportunities in the appropriate supplier selection process. The rest of this paper is divided into five parts. Section 2 introduces the literature review and methodology, and Section 3 presents construction steps of entropy-AHP weighted TOPSIS. Section 4 shows a numerical execution example of building material supplier selection. Section 5 is for results and discussion. Finally, Section 6 presents the conclusions of the study.

## 2. Literature Review and Methodology

**Figure 1:**
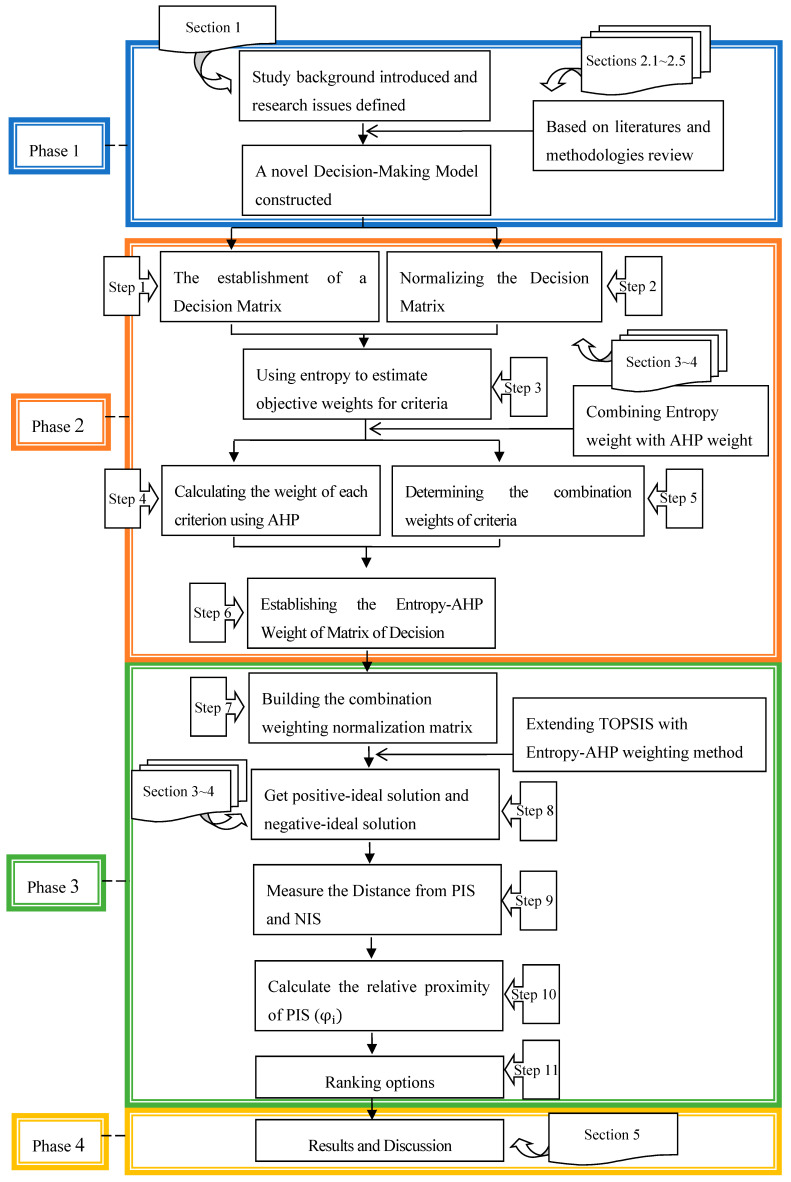
Research framework and analytic processes of sections and steps.

The analytic processes of the research framework consist of four stages, as shown in Figure 1:

Phase 1: Introduce the research background and review literatures and methods.

Phase 2: Begin to establish a novel TOPSIS model and then combine this model with the entropy and AHP weights.

Phase 3: Extending the TOPSIS method with the entropy-AHP weighting method.

Phase 4: Results and discussion.

Section 2.1, Section 2.2, Section 2.3, Section 2.4 and Section 2.5 are described in Phase 1, including a review of relevant literatures and methodologies, such as TOPSIS, the entropy weighted method, the AHP method, the combination weighting method and the weights for multi-criteria decision making. As for Section 3, Section 4 and Section 5, it belongs to other phases. Section 6 concludes this study.

### 2.1. Literature Review

#### 2.1.1. Literature on the Application of the Entropy, AHP, and/or TOPSIS Method

This section explores related literature on the Entropy, AHP, and/or TOPSIS method studied by researchers. Information entropy is derived from information theory [33], which was originally applied to assess the uncertainty of hydrological models [34]. The experiments show that the entropy information significantly improves the recognition rate and the robustness of the algorithm [35]. Analyzing the coordination relationship between economic development and investment potential with methods such as the entropy weight method, etc. [36]. Based on using TOPSIS technology and applying entropy weight information to calculate criteria weights, aim of this study is to select suitable suppliers in a green environment [37]. Using TOPSIS and entropy methods, a simulation-based multi-objective evaluation of a flow corridor is constructed [38].

By a comparative analysis of AHP and TOPSIS technologies, we can select the optimal transfer station location in Istanbul, Turkey [39]. Based on applying the AHP and TOPSIS methods under a fuzzy environment, the best type of dam spillway in Northern Greece can be selected [40]. On the basis of fuzzy AHP and TOPSIS methods, it allows us to evaluate suppliers and developing mathematical techniques to allocate orders [41]. The social, technical, economical, environmental, and political (STEEP) fuzzy AHP-TOPSIS framework is applied to select the location most suitable for Indian thermal power plants. The fuzzy AHP is used to determine the weights of the quantitative and qualitative criteria affecting the location selection process [42]. Combining neutrosophic (N) theory with AHP and TOPSIS methods to deal with uncertainty, ambiguity, and complexity [43]. Neutrosophic sets, which have been integrated with other technologies, such as AHP and the TOPSIS recently, are used to solve problems [44]. TOPSIS and ELECTRE I models are applied to the diagnostic fuzzy information of medicine [45]. A Delphi-DEMATEL-ANP-TOPSIS hybrid model was established to select strategies for dealing with derailment risks [46].

Based on the above literatures, the research methods for constructing models and solving problems were proposed.

#### 2.1.2. Rank Reversals in Decision-Making

In a multi-criteria decision-making process, ranking reversal refers to a change in ranking that overrides the order of possible decisions when, for example, a selection change method or a set of other available alternatives changes. The problem of ranking reversal is at the core of many debates in decision making and multi-criteria decision making. The following multi-criteria decision-making methods have been proven to exhibit various types of ranking reversals, such as [47,48,49,50,51,52]:

1. Multi-attribute utility theory (MAUT).
2. The TOPSIS method.
3. The analytic hierarchy process (AHP) and some of its variants.
4. The ELECTRE (outranking) method and its variants.
5. The PROMETHEE (outranking) method.

Because of the existence of rank reversals, which were described above, the limitation of the novel multi-criteria decision-making model, which is a mix of AHP and TOPSIS may also exist.

### 2.2. Entropy Weighted Method

#### 2.2.1. Entropy Weight Principle

Entropy is a thermodynamic concept proposed by German physicist R. Clausius in 1865. It describes the disorder or chaos of a thermodynamic system and is a state parameter of matter. In 1948, Shannon introduced entropy into information theory, which measures the uncertainty of signals in information sources, called information entropy.

The entropy weight method mainly uses the magnitude of the entropy value in information theory to represent the uncertainty of the message, calculates the ability of each evaluation attribute to pass decision information, and calculates the relative weight between attributes. From the judgment matrix, the entropy weight can be calculated [53,54,55,56]. “The smaller the entropy of the evaluated information criterion, the greater the weight of the information criterion”. This is only true if the underlying assumptions that all sources of information are reliable. Of course, the novel multi-criteria decision-making model proposed in this paper is no exception.

#### 2.2.2. Significance and Nature of Entropy Weight Method

Th method of the entropy weight is to calculate the information entropy of the indicator and use the degree of difference of the indicator to measure the effective information and indicator weight contained in the known data. The entropy weight indicates the relative importance coefficient of each indicator in the competition under the conditions of a given evaluation object and evaluation index when making a decision or evaluation plan, but it does not indicate the importance coefficient of the indicator in the practical sense. Its properties are as follows:

- If the values of elements in a column are the same, the maximum entropy is 1, and the entropy weight is 0. On behalf of an indicator, if the data of each evaluation object are the same, the indicator does not contain any valuable information.
- The greater the difference between the values of elements in a column, the smaller the entropy value of the elements in this column and the larger the entropy weight value. It indicates that the indicator has valuable information. Conversely, if the indicator’s entropy value is larger, the smaller its entropy weight and the less important this indicator is.

The basic calculation steps are as follows:

1. Initial data matrix normalization

Set m evaluation objects and n evaluation indicators to form the initial data matrix of the entropy weight evaluation system.

(1) $$X\;=\;\left[\begin{array}{cccc} x_{11} & x_{12} & \ldots & x_{1n}\\ x_{21} & x_{22} & \ldots & x_{2n}\\ \vdots & \vdots & & \vdots \\ {\;x}_{m1} & x_{m2} & \ldots & x_{\mathrm{mn}} \end{array}\right]\;=\;(X_{1\;\;}X_{2\;\;}\ldots \;X_{n\;\;})$$

where $x_{\mathrm{ij}}\left(i=1,\;2,\;\ldots ,\;m;j=1,\;2,\ldots ,\;n\right)$ represents the value of the i-th evaluation alternative in the j-th indicator, and $X_{j\;}$(j = 1, 2,…, n) denotes the column vector data of all evaluation alternatives of the j-th indicator.

Due to the differences in the indicator units, for the purpose of eliminating the impact of different units on the evaluation results, each indicator needs to be standardized. The step transformation method is often used and the formula is:

(2) $$\begin{array}{c} x_{\mathrm{ij}}^{\prime }=\frac{x_{\mathrm{ij}-}\underset{i}{\min}\left\{x_{\mathrm{ij}}\right\}}{\underset{i}{\max}\left\{x_{\mathrm{ij}}\right\}-\underset{i}{\min}\left\{x_{\mathrm{ij}}\right\}}\\ \mathrm{or}\;x_{j}^{\prime }=\frac{x_{\mathrm{ij}-}\underset{i}{\min}\left\{x_{\mathrm{ij}}\right\}}{\underset{i}{\max}\left\{X_{j}\right\}-\underset{i}{\min}\left\{X_{j}\right\}}\;(\mathrm{applicable}\;\mathrm{benefit}\;\mathrm{indicators}) \end{array}$$

(3) $$\begin{array}{c} x_{\mathrm{ij}}^{\prime }=\frac{\underset{i}{\max}\left\{x_{\mathrm{ij}}\right\}-x_{\mathrm{ij}}}{\underset{i}{\max}\left\{x_{\mathrm{ij}}\right\}-\underset{i}{\min}\left\{x_{\mathrm{ij}}\right\}}\\ \mathrm{or}\;x_{j}^{\prime }=\frac{\underset{i}{\max}\left\{x_{\mathrm{ij}}\right\}-x_{\mathrm{ij}}}{\underset{i}{\max}\left\{X_{j}\right\}-\underset{i}{\min}\left\{X_{j}\right\}}\;(\mathrm{applicable}\;\cos t\;\mathrm{indicators}) \end{array}$$

2. Estimate the proportion $z_{\mathrm{ij}}$ of the j-th indicator i-th evaluation alternative $x_{\mathrm{ij}}^{\prime }$

(4) $$z_{\mathrm{ij}}=\frac{x_{\mathrm{ij}}^{\prime }}{\sum_{i=1}^{m}x_{\mathrm{ij}}^{\prime }}\;\mathrm{or}\;Z_{j}=\frac{X_{j}^{\prime }}{\sum^{\text{​}}X_{j}^{\prime }}\;(j\;=\;1,\;2,\ldots ,\;n)$$

So, get the weight matrix:

(5) $$Z\;=\;{\left(z_{\mathrm{ij}}\right)}_{m\times n}\;\mathrm{or}\;Z\;=\;(Z_{1}Z_{2}\ldots {\;Z}_{n\;})$$

3. Calculate the value of the information entropy of the j-th indicator $e_{j}$

(6) $$e_{j}=-\pi \sum_{i=1}^{m}z_{\mathrm{ij}}\ln z_{\mathrm{ij}}\;(j\;=\;1,\;2,\ldots ,\;n)\;$$

among them, $\pi =\frac{1}{\ln m}$ is non-negative constant, and $0\leq e_{j}\leq 1$. When set $z_{\mathrm{ij}}=0$, $z_{\mathrm{ij}}\ln z_{\mathrm{ij}}$ = 0.

4. Calculate the information utility $b_{j}$ for the j-th indicator

(7) $$b_{j}\;=\;1-e_{j}\;(j\;=\;1,\;2,\ldots ,\;n)\;$$

5. Calculate the weight $w_{j}\;$ of the j-th indicator

(8) $$w_{\mathrm{ej}}=\frac{b_{j}}{\sum_{j=1}^{n}b_{j}}=\frac{1-e_{j}}{n-\sum_{j=1}^{n}e_{j}}\;(j\;=\;1,\;2,\ldots ,\;n)\;$$

6. Estimate the evaluation value $U_{i}$ of the evaluation plan i

(9) $$U_{i}=\sum_{j=1}^{n}z_{ij}w_{ej}\;(i\;=\;1,\;2,\ldots ,\;m)\;$$

When the entropy value is smaller, the degree of dispersion of the indicator is greater. It shows that the greater the usefulness of the information, the larger the impact of this indicator on the target in the comprehensive evaluation.

### 2.3. AHP Method

#### 2.3.1. The Meaning of AHP

The Analytic Hierarchy Process (AHP) method was first created in 1971 by Professor Thomas L. Saaty of the University of Pittsburgh. The AHP method reduces the complex problem system to a clear element system. In the analysis process, the eigenvector is used to represent the priority ratio between the elements in a hierarchy, and then the eigenvalue is obtained as the basis for evaluating the consistency of the dual comparison matrix performed on the nominal scale. If the consistency conditions are met, the priority order represented by the eigenvector will be used as the basis for selection or decision making [57,58,59].

#### 2.3.2. Application of AHP

The objective hierarchy structure can be constructed by hierarchical analysis. The methods that can be adopted to develop the objective hierarchy structure are generally related literature review, systematic analysis, and empirical analysis. The weighting system of the hierarchical analysis method, that is, by the experts and scholars’ understanding of the evaluation system, the evaluation items and criteria are made into a pairwise ratio allocation on a category scale. Since the weight obtained by the AHP method is obtained through the recognition of experts and scholars, it is classified as one of the subjective weights.

In other words, every two factors are compared in pairs and a dual comparison reciprocal matrix is established to obtain the feature vector. This feature vector is the relative weight of each factor and the relative importance in a particular category. Then, the eigenvalues are obtained from the eigenvectors. After the consistency check of the dual matrix, the conformance of this matrix can be accepted. The consistency test can find that in some questionnaires, when the dual comparison is performed, the C factor is less important than the B factor and the B factor is less important than the A factor. Contradictory questionnaires can be tested by a consistency check. Additionally, through the questionnaire, the eigenvector can be used as the basis for evaluating the weight.

#### 2.3.3. Steps of AHP Method

The AHP method proceeds as follows:

1. Build a hierarchical structure

**Figure 2:**
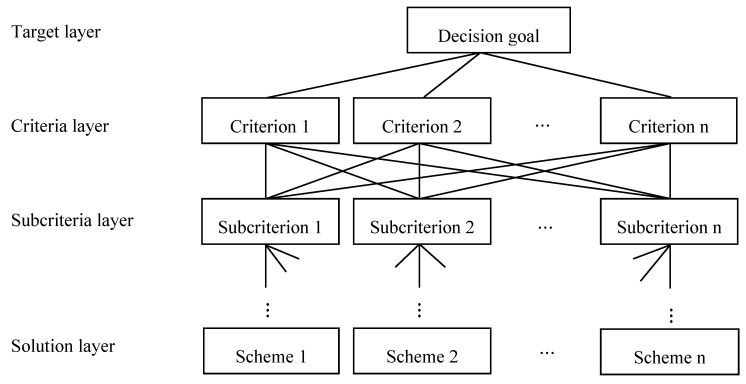
Schematic diagram of the hierarchy.

Classify the factors involved in the problem and construct a hierarchical structure of the interconnections between the factors. A typical hierarchical structure is shown in Figure 2.

2. Calculate the weight of each single-level model

3. Calculate the combined weight of each level element

The combined weights of the hierarchical elements can be calculated according to Table 1, obviously $\sum_{j=1}^{n}s_{j}=1$.

4. Evaluate the overall ranking of the hierarchy and calculate the consistency of the results

There are two main AHP consistency tests: one is the consistency index (CI) test, and the other is the consistency ratio (CR) test.

(1) CI test

CI means (λmax-n)/(n-1) where the closer the value of λmax is to n, the better the consistency and the smaller the CI value.

(2) CR test

CR means CI/RI where RI is a consistency and random index that randomly generates a reciprocal matrix, and is affected by the order. When the order n is larger, the value increases. Saaty (1981) believes that when the value of the consistency ratio (CR) is equal to or less than 0.1, the consistency of this matrix is acceptable.

### 2.4. Combination Weighting Method

The method of comprehensively considering the subjective weight and the objective weight of the evaluation standard is the combination weighting method. In the “multi-criteria decision” evaluation method, the weight value of the criteria has the ability to affect the evaluation result and has an important influence on the choice of the scheme. The combined weighting method will reduce the possible bias of a single subjective or objective weight on the choice of scheme. Suppose there are n evaluation index items at a certain level or an evaluation facet.

The weights determined by the AHP method and the entropy weight method are $\;W_{h}=\left(w_{h1},w_{h2},\ldots ,w_{hn}\right)$ and $W_{e}=\left(w_{e1},w_{e2},\ldots ,w_{\mathrm{en}}\right)$. The calculation of a combination weight that integrating the subjective weights and objective weights of the n criteria, is expressed as follows:

(10) $$w_{cj}=\frac{w_{ej}\times w_{hj}}{\sum_{j=1}^{n}w_{ej}\times w_{hj}},\;j=\;1,\;2,\ldots ,\;n.$$

### 2.5. Weights for Multi-Criteria Decision Making

In the evaluation method of multi-criteria decision making, the criterion weight often has a great influence on the choice of the scheme. That is, different criterion weights may lead to different evaluation results. Generally speaking, the calculation methods of criterion weights can be divided into the following three categories:

1. Subjective weight: 

Weights based on the subjective consciousness of decision makers or experts. In addition to the direct calculation of subjective weights by decision makers, many scholars have developed other methods, such as: (1) the AHP method [60]; (2) the extreme weight approach [61];(3) the weighted least square method [62]; (4) the linear programming techniques for multidimensional analysis of preference (LINMA) method [63].

The timing of applying subjective weights may lie in the evaluation of quantification and qualitative criteria, especially when expert expertise is required.

2. Objective weight: 

Weights based on evaluation matrix measurements. The calculation methods of objective weights include: (1) th entropy weight method [28]; (2) the gray relational matrix method [64].

The timing of applying objective weights can only be in the evaluation of quantitative criteria, and the sources of quantitative data should be reliable. This important basic assumption is necessary.

3. Eclectic weights: 

The weights are calculated based on the combination weighting method. Its purpose is to consider both the subjective weights of the decision makers and the objective weights calculated by the entropy weight method through the measurement of each criterion. And its advantages are [65]:

(1) The difference between subjective and objective weights can be compromised.

(2) The deviation of the evaluation results can be reduced.

(3) High reliability of analysis results.

## 3. Construction Steps of Entropy-AHP Weighted TOPSIS 

Let the set of alternatives be expressed as A = $\left\{A_{1},A_{2},\ldots ,A_{m}\right\}$, and the set of criteria be expressed as C = $\left\{C_{1},C_{2},\ldots ,C_{n}\right\}$, the construction process of entropy-AHP weighted TOPSIS method can be shown as follows:

Step 1: Constructing the decision matrix.

In this step, we obtain performance value $d_{\mathrm{ij}}$ and establish the decision matrix D = ${\left[d_{\mathrm{ij}}\right]}_{m\times n}$. The decision matrix for evaluation can be presented as follows:

(11) $$D=\;\begin{array}{cc} & \begin{array}{ccc} C_{1} & C_{2} & \begin{array}{cc} \;\ldots & \;C_{n} \end{array} \end{array}\\ \begin{array}{c} A_{1}\\ A_{2}\\ \begin{array}{c} \vdots \\ A_{m} \end{array} \end{array} & \left[\begin{array}{ccc} d_{11} & d_{12} & \begin{array}{cc} \ldots & d_{1n} \end{array}\\ d_{21} & d_{22} & \begin{array}{cc} \ldots & d_{2n} \end{array}\\ \begin{array}{c} \vdots \\ d_{m1} \end{array} & \begin{array}{c} \vdots \\ d_{m2} \end{array} & \begin{array}{cc} \begin{array}{c} \ddots \\ \ldots \end{array} & \begin{array}{c} \vdots \\ d_{mn} \end{array} \end{array} \end{array}\right] \end{array}$$

The decision matrix D contains n criteria and m alternatives. In addition, C denotes the criterion and A denotes the alternative.

Step 2: Normalizing the decision matrix.

In order to make utility preferences have a consistent unit of measurement, while avoiding extreme values that affect the measurement of similarity distances, statistical normalization methods are used to normalize performance values. A normalized performance value ($p_{\mathrm{ij}}$) is expressed as follows:

(12) $$p_{\mathrm{ij}}=\frac{d_{\mathrm{ij}}}{\sum_{i}d_{\mathrm{ij}}},\forall i,\;j$$

Then novel decision matrix can be expressed as follows:

(13) $$D^{*}=\begin{array}{cc} & \begin{array}{ccc} C_{1} & {\;C}_{2}\; & \;\begin{array}{cc} \ldots & {\;C}_{n} \end{array} \end{array}\\ \begin{array}{c} A_{1}\\ A_{2}\\ \begin{array}{c} \vdots \\ A_{m} \end{array} \end{array} & \left[\begin{array}{ccc} p_{11} & p_{12} & \begin{array}{cc} \ldots & p_{1n} \end{array}\\ p_{21} & p_{22} & \begin{array}{cc} \ldots & p_{2n} \end{array}\\ \begin{array}{c} \vdots \\ p_{m1} \end{array} & \begin{array}{c} \vdots \\ p_{m2} \end{array} & \begin{array}{cc} \begin{array}{c} \ddots \\ \ldots \end{array} & \begin{array}{c} \vdots \\ p_{\mathrm{mn}} \end{array} \end{array} \end{array}\right] \end{array}$$

Step 3: Calculating the objective weights of criteria using the entropy method.

The value ($e_{j}$) for the information entropy of the j-th criterion can be calculated as follows:

(14) $$e_{j}=-\pi \sum_{i=1}^{m}z_{\mathrm{ij}}\ln z_{\mathrm{ij}}\;(j\;=\;1,\;2,\ldots ,\;n)\;$$

where $z_{\mathrm{ij}}=\frac{p_{\mathrm{ij}}}{\sum_{i=1}^{m}p_{\mathrm{ij}}}$, $\pi =\frac{1}{\ln m}$ is a non-negative constant, and $0\leq e_{j}\leq 1$. Then, calculate the weight $w_{\mathrm{ej}}\;$ of the j-th index.

(15) $$w_{\mathrm{ej}}=\frac{1-e_{j}}{n-\sum_{j=1}^{n}e_{j}}\;(j\;=\;1,\;2,\ldots ,\;n)\;$$

Step 4: Calculating the weights of criteria using the AHP method.

According to the steps of the 2.2.3 AHP method. The AHP weight of each criterion can be obtained as follows:

(16) $$w_{hj}=\left(w_{h1},w_{h2},\ldots ,w_{hn}\right)\;(j\;=\;1,\;2,\ldots ,\;n)\;$$

Step 5: Determining the combination weight of each criterion. 

According to Equation (15) and (16), the weight of each criterion can be obtained on the basis of the combined weighting method as follows. The weight of each criterion is determined by a combined weighting method.

(17) wicj=wiej×wihj∑j=1nwiej×wihj, i = 1, 2,…, m; j = 1, 2,…, n 

where $w_{ej}$ is the objective weight derived from entropy and $w_{hj}$ is the subjective weight derived from the AHP method.

Step 6: Establishing the entropy-AHP weight of the decision matrix.

The entropy-AHP weight of the decision matrix can be expressed as follows:

(18) wcj =w1ci×w2cj, i = 1, 2,…, m; j = 1, 2,…, n 

Step 7: Building the combination weighting normalization decision matrix.

In order to reflect the fact that the greater the weight of the evaluation criterion, the more important its performance value is. The performance value of the criterion must be multiplied by the weight. The combination weighting normalization decision matrix can be expressed as follows:

(19) $$u\;=\;\begin{array}{cc} & \begin{array}{ccc} C_{1} & {\;C}_{2}\; & \;\begin{array}{cc} \ldots & {\;C}_{n} \end{array} \end{array}\\ \begin{array}{c} A_{1}\\ A_{2}\\ \begin{array}{c} \vdots \\ A_{m} \end{array} \end{array} & \left[\begin{array}{ccc} u_{11} & u_{12} & \begin{array}{cc} \ldots & u_{1n} \end{array}\\ u_{21} & u_{22} & \begin{array}{cc} \ldots & u_{2n} \end{array}\\ \begin{array}{c} \vdots \\ u_{m1} \end{array} & \begin{array}{c} \vdots \\ u_{m2} \end{array} & \begin{array}{cc} \begin{array}{c} \ddots \\ \ldots \end{array} & \begin{array}{c} \vdots \\ u_{\mathrm{mn}} \end{array} \end{array} \end{array}\right] \end{array}$$

(20) $$\mathrm{where}\;u_{ij}=w_{cj}\times p_{ij},\;\forall i,j$$

Step 8: Acquire the solutions of the negative-ideal (NI) and the positive-ideal (PI).

The evaluation criteria of the TOPSIS method can be divided into cost criteria and benefit criteria. Let C be a set of cost criteria and B be a set of benefit criteria. $A^{-}$ represents the negative-ideal solution and $A^{+}$ is the positive-ideal solution. Then, $A^{+}$ and $A^{-}$ can be acquired as:

(21) $$\begin{array}{l} A^{+}=\left(\;\left(\underset{i}{\max}\mu_{\mathrm{ij}}|\;j\in B\right),\;\left(\underset{i}{\min}\mu_{\mathrm{ij}}|\;j\in C\right)\right)\\ \;=\left(u_{j}^{+}|\;j=1,2,\ldots ,m\right) \end{array}$$

(22) $$\begin{array}{l} A^{-}=\left(\;\left(\underset{i}{\min}\mu_{\mathrm{ij}}|\;j\in B\right),\;\left(\underset{i}{\max}\mu_{\mathrm{ij}}|\;j\in C\right)\right)\\ \;=\left(u_{j}^{-}|\;j=1,2,\ldots ,m\right) \end{array}$$

Step 9: Measure the distance from NIS (solution of NI) and PIS (solution of PI).

For the purpose of calculating the distance from PIS or NIS to each alternative $A_{i}$. The Euclidean distance is expressed by the following calculation:

(23) $$S^{+}=\sqrt{\overset{n}{\underset{j=1}{\sum }}{\left(u_{\mathrm{ij}}-u_{\mathrm{ij}}^{+}\right)}^{2}}$$

(24) $$S^{-}=\sqrt{\overset{n}{\underset{j=1}{\sum }}{\left(u_{\mathrm{ij}}-u_{\mathrm{ij}}^{-}\right)}^{2}}$$

Step 10: Calculate the relative proximity of PIS. 

The relative proximity of an alternative $A_{i}$ to the positive-ideal solution (PIS) $A^{+}$ can be expressed as follows:

(25) $$\varphi_{i}=\frac{S^{-}}{S^{+}+S^{-}},\;\mathrm{where}\;0\leq \varphi_{i}\leq 1$$

Step 11: Ranking the alternatives. 

Alternatives are ranked in descending order of $\varphi_{i}$’s value according to the relative proximity of each alternative. Some alternatives have larger relative proximity values, indicating that they are closer to PIS. Finally, the most suitable choice will be the one with the highest proximity value.

## 4. Numerical Execution Example of Building Material Supplier Selection

A capital company wants to select the most appropriate supplier as its investment target. Five building material suppliers were selected as alternatives for further evaluation. The numerical input values are quoted or revised by referring to the data of some building material suppliers in Taiwan. To avoid unnecessary commercial disputes, the supplier’s names have not been disclosed. Although the supplier name is represented by the codes A1~A5, this representation will not affect the process and results of the novel multi-criteria decision-making model in selecting the appropriate supplier.

**Figure 3:**
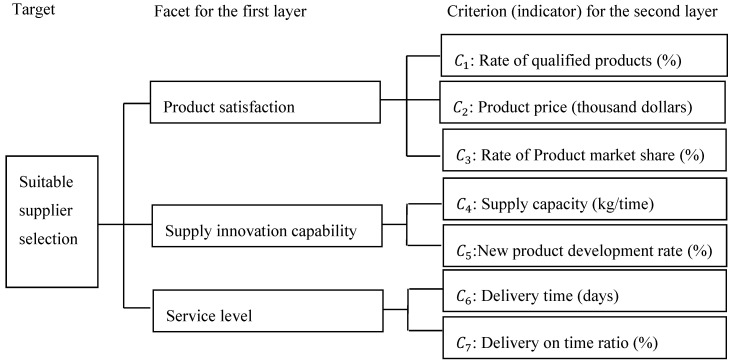
Hierarchical analysis diagram of this study.

The first layer contains 3 facets of product satisfaction, supply innovation capability, and service level. The second layer includes 7 criteria, such as *C*_1_: the rate of qualified products (%), *C*_2_: the product price (thousand dollars), *C*_3_: the rate of product market share (%), *C*_4_: the supply capacity (kg/time), *C*_5_: the new product development rate (%), *C*_6_: the delivery time (days), and *C*_7_: the delivery on time ratio (%). The analytic hierarchy diagrams of the two levels and the seven criteria are shown in Figure 3.

Step 1: Constructing the decision matrix.

A decision matrix is established with five alternatives and seven criteria. The decision matrix was expressed as below:

$$D=\begin{array}{cc} & \begin{array}{ccc} C_{1} & C_{2} & \begin{array}{cc} \ldots & C_{7} \end{array} \end{array}\\ \begin{array}{c} A_{1}\\ A_{2}\\ \begin{array}{c} \vdots \\ A_{5} \end{array} \end{array} & \left[\begin{array}{ccc} d_{11} & d_{12} & \begin{array}{cc} \ldots & d_{17} \end{array}\\ d_{21} & d_{22} & \begin{array}{cc} \ldots & d_{27} \end{array}\\ \begin{array}{c} \vdots \\ d_{51} \end{array} & \begin{array}{c} \vdots \\ d_{52} \end{array} & \begin{array}{cc} \begin{array}{c} \ddots \\ \ldots \end{array} & \begin{array}{c} \vdots \\ d_{57} \end{array} \end{array} \end{array}\right] \end{array}=\begin{array}{cc} & \begin{array}{lllllll} \;C_{1} & \;C_{2} & \;C_{3} & \;C_{4} & \;C_{5} & C_{6} & \;C_{7} \end{array}\\ \begin{array}{c} A_{1}\\ A_{2}\\ A_{3}\\ A_{4}\\ A_{5} \end{array} & \left[\begin{array}{ccccccc} 0.95 & 36 & 0.19 & 53 & 0.73 & 11 & 0.93\\ 0.98 & 39 & 0.17 & 52 & 0.75 & 13 & 0.89\\ 0.93 & 33 & 0.21 & 57 & 0.69 & 11 & 0.92\\ 0.91 & 37 & 0.23 & 56 & 0.77 & 12 & 0.87\\ 0.92 & 35 & 0.16 & 51 & 0.76 & 10 & 0.86 \end{array}\right] \end{array}$$

where A represents the alternative and C represents the criterion. 

Step 2: Normalizing the decision matrix.

In this step, the new decision matrix based on normalized performance by Equation (12) $p_{\mathrm{ij}}=\frac{d_{\mathrm{ij}}}{\sum_{i}d_{\mathrm{ij}}}\;$ and Equtation (13) is as follows:

$$D^{*}=\begin{array}{cc} & \begin{array}{ccc} C_{1} & {\;C}_{2}\; & \;\begin{array}{cc} \ldots & {\;C}_{7} \end{array} \end{array}\\ \begin{array}{c} A_{1}\\ A_{2}\\ \begin{array}{c} \vdots \\ A_{5} \end{array} \end{array} & \left[\begin{array}{ccc} p_{11} & p_{12} & \begin{array}{cc} \ldots & p_{17} \end{array}\\ p_{21} & p_{22} & \begin{array}{cc} \ldots & p_{27} \end{array}\\ \begin{array}{c} \vdots \\ p_{51} \end{array} & \begin{array}{c} \vdots \\ p_{52} \end{array} & \begin{array}{cc} \begin{array}{c} \ddots \\ \ldots \end{array} & \begin{array}{c} \vdots \\ p_{57} \end{array} \end{array} \end{array}\right] \end{array}=\begin{array}{cc} & \begin{array}{lllllll} \;C_{1} & \;C_{2} & \;C_{3} & \;C_{4} & \;C_{5} & \;C_{6} & \;C_{7} \end{array}\\ \begin{array}{c} A_{1}\\ A_{2}\\ A_{3}\\ A_{4}\\ A_{5} \end{array} & \left[\begin{array}{ccccccc} 0.2026 & 0.2000 & 0.1979 & 0.1970 & 0.1973 & 0.1930 & 0.2081\\ 0.2090 & 0.2167 & 0.1771 & 0.1933 & 0.2027 & 0.2281 & 0.1991\\ 0.1983 & 0.1833 & 0.2188 & 0.2119 & 0.1865 & 0.1930 & 0.2058\\ 0.1940 & 0.2056 & 0.2396 & 0.2082 & 0.2081 & 0.2105 & 0.1946\\ 0.1962 & 0.1944 & 0.1677 & 0.1896 & 0.2054 & 0.1745 & 0.1924 \end{array}\right] \end{array}$$

Step 3: Calculating the objective weights of criteria using the entropy method.

According to Figure 3, the analysis group can be divided into 4 categories. Category 1 is “product satisfaction” (3 criteria), Category 2 is “supply innovation capability” (2 criteria), Category 3 is “service level” (2 criteria), and Category 4 is “suitable supplier selection” (7 criteria). This step will use Category 1 as a case to demonstrate the calculation process of its entropy weight. 

(1) Initial data matrix normalization.

Based on the decision matrix in Step 1, set 5 alternatives and 3 criteria to form the initial data matrix of the evaluation system of Category 1.

$$D_{E1}=\begin{array}{cc} & \begin{array}{ccc} C_{1} & C_{2} & \begin{array}{c} C_{3} \end{array} \end{array}\\ \begin{array}{c} A_{1}\\ A_{2}\\ \begin{array}{c} \vdots \\ A_{5} \end{array} \end{array} & \left[\begin{array}{ccc} d_{11} & d_{12} & \begin{array}{c} d_{13} \end{array}\\ d_{21} & d_{22} & \begin{array}{c} d_{23} \end{array}\\ \begin{array}{c} \vdots \\ d_{51} \end{array} & \begin{array}{c} \vdots \\ d_{52} \end{array} & \begin{array}{c} \begin{array}{c} \vdots \\ d_{53} \end{array} \end{array} \end{array}\right] \end{array}=\begin{array}{cc} & \begin{array}{ccc} C_{1} & C_{2} & C_{3} \end{array}\\ \begin{array}{c} A_{1}\\ A_{2}\\ A_{3}\\ A_{4}\\ A_{5} \end{array} & \left[\begin{array}{ccc} 0.95 & 36 & 0.19\\ 0.98 & 39 & 0.17\\ 0.93 & 33 & 0.21\\ 0.91 & 37 & 0.23\\ 0.92 & 35 & 0.16 \end{array}\right] \end{array}$$

Because $C_{1}\;$ is a benefit criterion, $C_{1}\;$’s element normalization applies to Equation (2) $x_{j}^{\prime }=\frac{x_{\mathrm{ij}-}\underset{i}{\min}\left\{x_{\mathrm{ij}}\right\}}{\underset{i}{\max}\left\{X_{j}\right\}-\underset{i}{\min}\left\{X_{j}\right\}}$. Taking $d_{11}^{\prime }$ as an example, the normalized calculation formula of $d_{11}^{\prime }$ is as follows:

$$d_{11}^{\prime }=\frac{d_{11-}\underset{1}{\min}\left\{d_{1j}\right\}}{\underset{1}{\max}\left\{d_{1j}\right\}-\underset{1}{\min}\left\{d_{1j}\right\}}=\frac{0.95-0.91}{0.98-0.91}=0.5714$$

In the same way, to calculate the normalized values for other elements of the $D_{E1}$ matrix, we obtain the matrix $D_{E1}^{\prime }$ as below:

$$D_{E1}^{\prime }=\begin{array}{cc} & \begin{array}{ccc} C_{1} & C_{2} & \begin{array}{c} C_{3} \end{array} \end{array}\\ \begin{array}{c} A_{1}\\ A_{2}\\ \begin{array}{c} \vdots \\ A_{5} \end{array} \end{array} & \left[\begin{array}{ccc} d_{11}^{\prime } & d_{21}^{\prime } & \begin{array}{c} d_{31}^{\prime } \end{array}\\ d_{12}^{\prime } & d_{22}^{\prime } & \begin{array}{c} d_{32}^{\prime } \end{array}\\ \begin{array}{c} \vdots \\ d_{15}^{\prime } \end{array} & \begin{array}{c} \vdots \\ d_{25}^{\prime } \end{array} & \begin{array}{c} \begin{array}{c} \vdots \\ d_{35}^{\prime } \end{array} \end{array} \end{array}\right] \end{array}=\begin{array}{cc} & \begin{array}{lll} \;C_{1} & \;C_{2} & \;C_{3} \end{array}\\ \begin{array}{c} A_{1}\\ A_{2}\\ A_{3}\\ A_{4}\\ A_{5} \end{array} & \left[\begin{array}{ccc} 0.5714 & 0.5000 & 0.4286\\ 1.0000 & 0.0000 & 0.1429\\ 0.2857 & 1.0000 & 0.7143\\ 0.0000 & 0.3333 & 1.0000\\ 0.1429 & 0.6667 & 0.0000 \end{array}\right] \end{array}$$

(2) Calculate the proportion $z_{\mathrm{ij}}$ of the j-th criterion i-th evaluation object $x_{\mathrm{ij}}^{\prime }$. 

According Equation (4) $z_{\mathrm{ij}}=\frac{x_{\mathrm{ij}}^{\prime }}{\sum_{i=1}^{m}x_{\mathrm{ij}}^{\prime }}$ and taking $z_{11}\;$ as an example, the proportion calculation formula of $z_{11}$ is as follows:

$$z_{11}=\frac{d_{11}^{\prime }}{\sum_{i=1}^{5}d_{i1}^{\prime }}=\frac{0.5714}{0.5714+1.0000+0.2857+0.0000+0.1429}=0.2857$$

In the same way, to calculate the proportion values for other elements of the $D_{E1}^{\prime }$ matrix, we obtain the matrix Z as below:

$$Z=\begin{array}{cc} & \begin{array}{ccc} C_{1} & C_{2} & \begin{array}{c} C_{3} \end{array} \end{array}\\ \begin{array}{c} A_{1}\\ A_{2}\\ \begin{array}{c} \vdots \\ A_{5} \end{array} \end{array} & \left[\begin{array}{ccc} z_{11} & z_{21} & \begin{array}{c} z_{31} \end{array}\\ z_{12} & z_{22} & \begin{array}{c} z_{32} \end{array}\\ \begin{array}{c} \vdots \\ z_{15} \end{array} & \begin{array}{c} \vdots \\ z_{25} \end{array} & \begin{array}{c} \begin{array}{c} \vdots \\ z_{35} \end{array} \end{array} \end{array}\right] \end{array}=\begin{array}{cc} & \begin{array}{lll} \;C_{1} & \;C_{2} & \;C_{3} \end{array}\\ \begin{array}{c} A_{1}\\ A_{2}\\ A_{3}\\ A_{4}\\ A_{5} \end{array} & \left[\begin{array}{ccc} 0.2857 & 0.2000 & 0.1875\\ 0.5000 & 0.0000 & 0.0625\\ 0.1429 & 0.4000 & 0.3125\\ 0.0000 & 0.1333 & 0.4375\\ 0.0714 & 0.2667 & 0.0000 \end{array}\right] \end{array}$$

(3) Calculate the value of the information entropy of the j-th criterion e_j_.

According Equation (6), $e_{j}=-\frac{1}{\ln m}\sum_{i=1}^{m}z_{\mathrm{ij}}\ln z_{\mathrm{ij}}$, taking e_1_ as an example, the proportion calculation formula of $e_{1}$ is as follows:

$$\begin{array}{c} e_{1}=-\frac{1}{\ln5}\sum_{i=1}^{5}z_{i1}\ln z_{i1}=-\frac{1}{\ln5}(0.2857\times \ln0.2857+0.5000\times \ln0.5000+0.1429\times \ln0.1429\\ +0.0000\times \ln0.0000+0.0714\times \ln0.0714)=0.7276 \end{array}$$

In the same calculation, we can obtain $e_{2}=$ 0.8137 and $e_{3}=$ 0.7533.

(4) Calculate the information utility $b_{j}$ for the j-th criterion.

According Equation (7) $b_{j}$ = 1 − $e_{j}$, the information utility $b_{j}$ for the j-th criterion can be calculated as follows:

b_1_ = 1 − e_1_ = 1 − 0.7276 = 0.2724; b_2_ = 1 − e_2_ = 1 − 0.8137 = 0.1863; b_3_ = 1 − e_3_ = 1 − 0.7533 = 0.2467

(5) Calculate the entropy weight $w_{j}$ of the j-th criterion. 

According Equation (8) $w_{\mathrm{ej}}=\frac{b_{j}}{\sum_{j=1}^{n}b_{j}}$, the entropy weight $w_{j}\;$ of the j-th criterion can be calculated as follows:

$$\begin{array}{c} w_{e1}=\frac{b_{1}}{\sum_{j=1}^{3}b_{j}}=\frac{0.2724}{0.2724+0.1863+0.2467}=0.3862\\ w_{e2}=\frac{b_{2}}{\sum_{j=1}^{3}b_{j}}=\frac{0.1863}{0.2724+0.1863+0.2467}=0.2641\\ w_{e3}=\frac{b_{3}}{\sum_{j=1}^{3}b_{j}}=\frac{0.2647}{0.2724+0.1863+0.2467}=0.3497 \end{array}$$

Following the same process, the entropy weights of the other three categories can also be calculated. So, the weights of the criterion and facet for evaluating building material supplier selection with the entropy method are expressed in Table 2.

The entropy weight (w1ei) of the facet can be obtained as follows:

w1ei=(w1e1,w1e2,w1e3)=(0.4426, 0.2592,0.2982)

The entropy weight (w2ej) of the criterion can be obtained as follows:

w2ej=(w2e1,w2e2,…,w2e7)=(0.3862, 0.2641,0.3497,0.4658,0.5342,0.4168,0.5832)

Step 4: Calculating the weight of each criterion using the AHP method.

Assuming that some decision makers and experts follow the steps of the 2.2.3 AHP method, the weights and indicators for evaluating various aspects of building material supplier selection with the AHP method are listed in Table 3.

The AHP weight (w1hi) of the facet in the first layer can be obtained as follows:

(w1hi)=(w1h1,w1h2,w1h3)=(0.3916, 0.2815,0.3269)

The AHP weights (w2hj) of criteria in second layer can be obtained as below:

w2hj=(w2h1,w2h2,…,w2h7)=(0.4125, 0.3759,0.2116,0.5293,0.4707,0.3917,0.6083)

Step 5: Determining the combination weights of the criteria. 

According to the Equation (17) $w_{cj}=\frac{w_{ej}\times w_{hj}}{\sum_{j=1}^{n}w_{ej}\times w_{hj}}$, where $w_{ej}$ is the objective weight calculated by entropy and $w_{hj}$ is the subjective weight derived from the AHP method, then the combination weight (w1ci)  and w2ci)  of each criterion can be obtained by using the combination weighting method, as expressed in Table 4 and Table 5.

The combination weight (w1ci) of the facet in first layer can be obtained as follows:

(w1ci)=(w1c1,w1c2,w1c3)=(0.5042, 0.2122,0.2836)

The combination weights (w2cj) of criteria in second layer can be obtained as below:

w2cj=(w2c1,w2c2,…,w2c7)=(0.4789, 0.2985,0.2225,0.4951,0.5049,0.3152,0.6848)

Step 6: Establishing the entropy-AHP weight of the decision matrix.

Based on Table 4 and Table 5, and by Equation (18), wcj =w1ci×w2cj, Table 6 showed the entropy-AHP weight ($w_{\mathrm{cj}}$) of the decision matrix.

We can obtain the entropy-AHP weight ($w_{cj}$) of each criterion using the equation listed below:

$$w_{cj}=\left(w_{c1},w_{c2},\ldots ,w_{c7}\right)=\left(0.2415,\;0.1505,0.1122,0.1051,0.1071,0.0894,0.1942\right)$$

Step 7: Building the combination weighting normalization decision matrix.

The combination weighting normalization decision matrix using Equation (19) and Equation (20), $u_{ij}=w_{cj}\times p_{ij},$ can be expressed as follows:

$$u\;=\;\begin{array}{cc} & \begin{array}{ccc} C_{1} & {\;C}_{2}\; & \;\begin{array}{cc} \ldots & {\;C}_{7} \end{array} \end{array}\\ \begin{array}{c} A_{1}\\ A_{2}\\ \begin{array}{c} \vdots \\ A_{5} \end{array} \end{array} & \left[\begin{array}{ccc} u_{11} & u_{12} & \begin{array}{cc} \ldots & u_{17} \end{array}\\ u_{21} & u_{22} & \begin{array}{cc} \ldots & u_{27} \end{array}\\ \begin{array}{c} \vdots \\ u_{51} \end{array} & \begin{array}{c} \vdots \\ u_{52} \end{array} & \begin{array}{cc} \begin{array}{c} \ddots \\ \ldots \end{array} & \begin{array}{c} \vdots \\ u_{57} \end{array} \end{array} \end{array}\right] \end{array}=\begin{array}{cc} & \begin{array}{lllllll} \;C_{1} & \;C_{2} & \;C_{3} & \;C_{4} & \;C_{5} & \;C_{6} & \;C_{7} \end{array}\\ \begin{array}{c} A_{1}\\ A_{2}\\ A_{3}\\ A_{4}\\ A_{5} \end{array} & \left[\begin{array}{ccccccc} 0.0489 & 0.0224 & 0.0298 & 0.0207 & 0.0211 & 0.0173 & 0.0404\\ 0.0505 & 0.0326 & 0.0267 & 0.0203 & 0.0217 & 0.0204 & 0.0387\\ 0.0479 & 0.0276 & 0.0329 & 0.0223 & 0.0200 & 0.0173 & 0.0400\\ 0.0468 & 0.0309 & 0.0361 & 0.0219 & 0.0223 & 0.0188 & 0.0378\\ 0.0474 & 0.0293 & 0.0251 & 0.0199 & 0.0220 & 0.0157 & 0.0374 \end{array}\right] \end{array}$$

Step 8: Acquire the solutions of the positive-ideal (PI) and the negative-ideal (NI).

The 7 criteria are classified as benefit criteria or cost criteria. “Rate of qualified products”, “Rate of Product market share”, “Supply capacity (kg/time)”, “New product development rate (%)”, and “Delivery on time ratio (%)” are benefit criteria B = {$C_{1}$,$C_{3}$,$C_{4},C_{5},C_{7}$}, however, “Product price (thousand dollars)” and “Delivery time (days)” are cost criteria C = {$C_{2}$,${\;C}_{6}$}. Then, we get the positive ideal (PI) and negative ideal (NI) solutions as follows:

$$A^{+}=\left\{0.0505,0.0224,0.0361,0.0223,0.0223,0.0157,0.0404\right\}$$

$$A^{-}=\left\{0.0468,0.0326,0.0251,0.0199,0.0200,0.0204,0.0374\right\}$$

Step 9: Measure the Euclidean distance from PI solution (PIS) and NI solution (NIS).

Based on the normalized Euclidean distance, distance measurement of both positive and negative solutions for each alternative by Equation (23) $S^{+}=\sqrt{\sum_{j=1}^{n}{\left(u_{\mathrm{ij}}-u_{\mathrm{ij}}^{+}\right)}^{2}}\;$ and Equation (24) $S^{-}=\sqrt{\sum_{j=1}^{n}{\left(u_{\mathrm{ij}}-u_{\mathrm{ij}}^{-}\right)}^{2}}\;$ are given in Table 7 and Table 8.

Step 10: Calculate the Relative Proximity of PIS.

**Figure 4:**
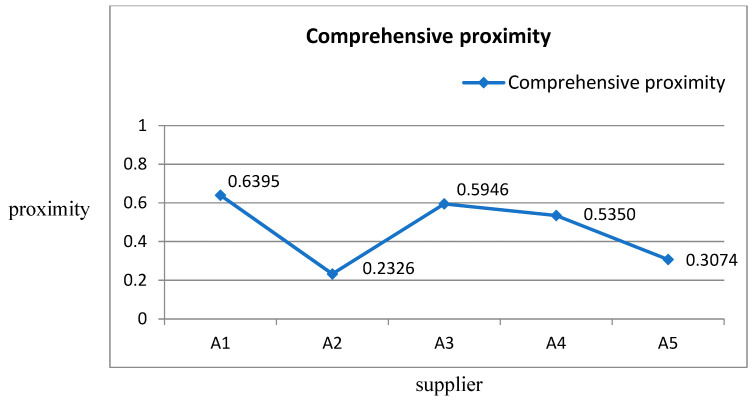
Comprehensive proximity of supplier alternatives.

Relative proximity of an alternative $A_{i}$ with regard to the positive-ideal solution (PIS) by Equation (25) $\varphi_{i}=\frac{S^{-}}{S^{+}+S^{-}}$ is shown in Table 9 and Figure 4. It can be seen from Figure 4 that the closer the proximity is to 1, the higher the overall performance of the supplier to be selected—that is, the most suitable supplier.

Step 11: Ranking the Options.

Based on the relative proximity of each alternative, and then options were ranked according to the descending order of $\varphi_{i}$. As shown in Table 10, the five options were ranked in order as $A_{1}$ > $A_{3}>A_{4}>A_{5}>A_{2}$. $A_{1}$ was selected as a suitable supplier of building materials in five alternatives.

## 5. Results and Discussion

In the multi-criteria evaluation method, a sensitivity analysis must be performed at the final stage in order to analyze the relationship between the weight of the alternatives and the proximity of TOPSIS. We will perform a sensitivity analysis and discuss the findings of this article in this section. From the 2nd to 4th stages of the research framework, the entropy-AHP weight value of TOPSIS can be obtained, which can appropriately replace the subjective weight value set by decision makers in the traditional TOPSIS method. The value of the obtained entropy-AHP weight vector is $w_{cj}=\left(w_{c1},w_{c2},\ldots ,w_{c7}\right)=(0.2415,\;0.1505,\;0.1122,\;0.1051,\;0.1071,\;0.0894,\;0.1942$). This means that the individual impact of each criterion on alternatives is 24.15%, 15.05%, 11.22%, 10.5%, 10.71%, 8.94%, and 19.42%, respectively. The combination of objective weight (entropy) and subjective weight (AHP) can reduce the bias of subjective weights and truly reflect the current situation. In the third phase, the TOPSIS method is improved by using the entropy-AHP weight, and a novel entropy-AHP weighted TOPSIS model is established.

In addition, based on the novel entropy-AHP TOPSIS model, the value of the evaluation index φ represents the relative advantage of each alternative. We can rank the alternatives in the order of A1, A3, A4, A5, and A2 according to the φ value from high to low. The suitable supplier was finally determined to be A1.

From the results, it can be seen that the research framework proposed in this paper has the advantage of choosing suitable alternatives and providing a reference value for decision makers. To verify the stability and robustness of the novel evaluation model, a systematic sensitivity analysis was performed and compared with the AHP-based TOPSIS model. 

According Table 4 and Table 5, the facets and indicator weights belonging to AHP-based TOPSIS and entropy-AHP TOPSIS can be shown as Table 11 and Table 12.

**Figure 5:**
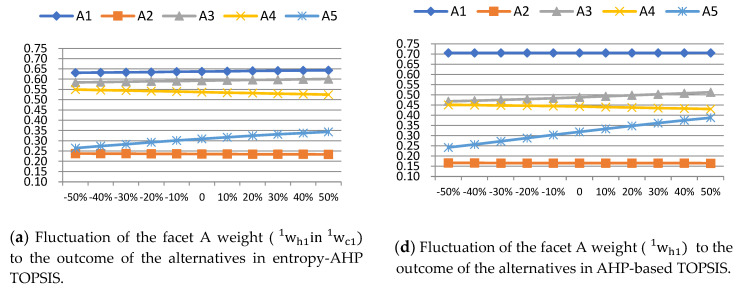

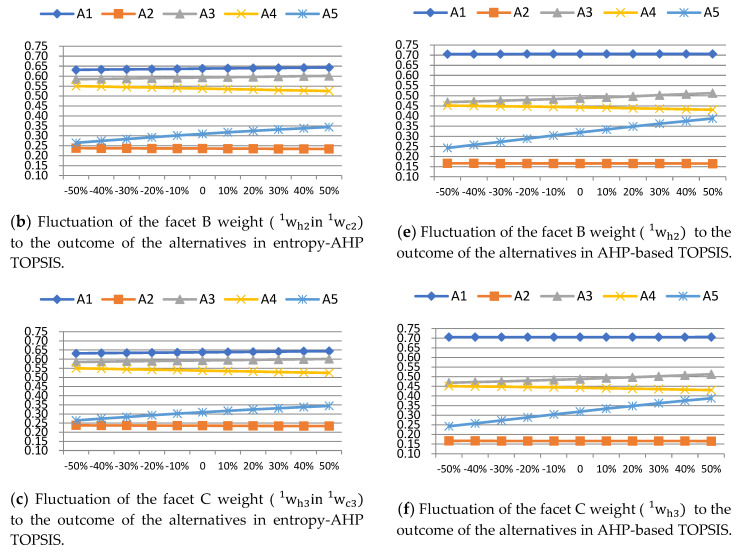
Sensitivity analysis of the facet weight to the outcome of the alternatives. ntropy-AHP TOPSIS vs. AHP-based TOPSIS.

First, explore the correspondence between the relative proximity of alternatives in entropy-AHP TOPSIS and AHP-based TOPSIS when the weights of facet A, B, C changes within the range of −50%, −40%, ..., 40%, 50%, shown as Table 13 and Figure 5. Based on this, it can be seen that the ranking of each alternative has not changed, indicating that the value of the facet weight does not affect the ranking of alternatives. 

And from the perspective of the best choice, no matter how the standard weight changes, the most suitable choice is still $A_{1}$.

Comparing the sensitivity analysis of the results of entropy-AHP TOPSIS [Figure 5a–c] and AHP-based TOPSIS [Figure 5d–f], we can know that ntropy-AHP TOPSIS is an effective and more stable evaluation model in selecting a building material supplier than AHP-based TOPSIS.

In summary, the sensitivity analysis proves that the evaluation results of the novel evaluation model established are valid and reliable. Through the sensitivity analysis of building material suppliers, the effectiveness, feasibility, and stability of the novel multi-criteria evaluation model for solving the MCDM problems are verified.

## 6. Conclusions

The aim of this research is to develop a novel entropy-AHP weighted TOPSIS model to evaluate the building material supplier selection from a theoretical and practical perspective. This paper describes how to comprehensively and systematically construct an entropy-AHP weighted TOPSIS method. From the suggested methods, Entropy, AHP, and TOPSIS are utilized to achieve research purposes. The findings and particular advantages of research indicate as follow:

The entropy-AHP weight value can be a suitable substitute for the weight value determined subjectively by decision makers in the TOPSIS method. Decision makers can more comprehensively and scientifically evaluate potential suppliers based on aggregating Entropy objective and AHP subjective weights into one comprised weight.

The combination of the weights between different layers of Entropy and AHP needs to be calculated separately. After combining the weights of each layer, the weights of different layers are then multiplied to obtain the total weight value (entropy-AHP weight).

Compared with the AHP-based TOPSIS model, the selection result of this novel evaluation model is effective, reliable and more stable.

In addition, throughout the research process and results, the contributions of this paper can mainly be expressed as below:

To combine entropy weight, AHP, and TOPSIS methods into a suitable MCDM solution. Provide effective information when decision makers are in an environment with insufficient information and strong subjective consciousness. 

With a compromise weight combining objective weight (Entropy weight) and subjective weight (AHP weight), to replace the subjective weight directly set by the decision maker in the TOPSIS method. To put it another way, using entropy-AHP weights instead of subjective weights can reduce biases that may be caused only by subjective and conscious judgments. 

To extend the TOPSIS method by entropy-AHP weights. That means the functionality of the TOPSIS method is extended based on the entropy-AHP weights. According to the combined weights, a normalized weight matrix and the relative proximity are calculated. Relative proximity values are used as the basis for proper supplier selection.

The theory and practice of the TOPSIS method based on entropy AHP weights have great opportunities for successful application in multi-criteria decision making because it complements and improves the subjective opinions of decision makers. The novel multi-criteria evaluation model can deal with related issues in the field of multi-criteria decision making, such as location selection, construction schemes, and many other disciplines governing decision making. The conclusions obtained in this study have taken us an important step forward, allowing us to use the Entropy-AHP weighted TOPSIS model more practically in the future.

## Figures and Tables

**Table 1:** Combined weights at each level.

		R-level	$R_{1}$	$R_{2}$	⋯	$R_{n}$	S-level element combination weight
	Weight		$r_{1}$	$r_{2}$	⋯	$r_{n}$	
S-level							
$S_{1}$			$s_{1}^{1}$	$s_{1}^{2}$	⋯	$s_{1}^{n}$	$s_{1}=\overset{n}{\underset{i=1}{\sum }}r_{i}s_{1}^{i}$
$S_{2}$			$s_{2}^{1}$	$s_{2}^{2}$	⋯	$s_{2}^{n}$	$s_{2}=\overset{n}{\underset{i=1}{\sum }}r_{i}s_{2}^{i}$
⋮			⋮	⋮		⋮	⋮
$S_{4}$			$s_{m}^{1}$	$s_{m}^{2}$	⋯	$s_{m}^{n}$	$s_{m}=\overset{n}{\underset{i=1}{\sum }}r_{i}s_{m}^{i}$

**Table 2:**
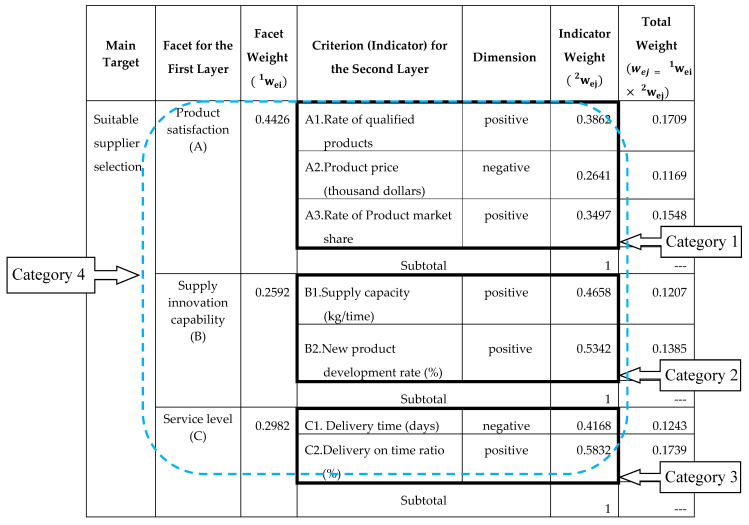
Weights of various facets and criteria for building material supplier selection evaluated with the entropy method.

**Table 3:** Weights of various facets and the criterion of building material supplier selection evaluated with the analytic hierarchy process (AHP) method.

Main Target	Facet for the First Layer	Facet Weight (w1hi)	Criterion (Indicator) for the Second Layer	Dimension	Indicator Weight (w2hj)	Total Weight (whj = w1hi×w2hj)
Suitable supplier selection	Product satisfaction (A)	0.3916	A1. Rate of qualified products	positive	0.4125	0.1615
			A2.Product price (thousand dollars)	negative	0.3759	0.1472
			A3.Rate of Product market share	positive	0.2116	0.0829
			Subtotal		1	---
	Supply innovation capability (B)	0.2815	B1.Supply capacity (kg/time)	positive	0.5293	0.1490
			B2.New product development rate (%)	positive	0.4707	0.1325
			Subtotal		1	---
	Service level (C)	0.3269	C1. Delivery time (days)	negative	0.3917	0.1280
			C2. Delivery on time ratio (%)	positive	0.6083	0.1989
			Subtotal		1	---

**Table 4:** Facet weights of building material supplier selection evaluated with the combination weighting method.

Weight Item	Product Satisfaction (A)	Supply Innovation Capability (B)	Service Level (C)
Entropy weight (w1ei)	0.4426	0.2592	0.2982
AHP weight (w1hi)	0.3916	0.2815	0.3269
Combination weight (w1ci=w1ei×w1hi∑i=13w1ei×w1hi)	0.5042	0.2122	0.2836

**Table 5:** Criterion weights of building material supplier selection evaluated by the combination weighting method.

Weight Item	Rate of Qualified Products (A1)	Product Price (Thousand Dollars) (A2)	Rate of Product Market Share (A3)	Supply Capacity (kg/ time) (B1)	New Product Development Rate (%) (B2)	Delivery Time (days) (C1)	Delivery on Time Ratio (%) (C2)
Entropy weight (w2ej)	0.3862	0.2641	0.3497	0.4658	0.5342	0.4168	0.5832
AHP weight (w2hj)	0.4125	0.3759	0.2116	0.5293	0.4707	0.3917	0.6083
Combination weight (w2cj=w2ej×w2hj∑j=17w2ej×w2hj)	0.4789	0.2985	0.2225	0.4951	0.5049	0.3152	0.6848

**Table 6:** The entropy-AHP weight ($w_{\mathrm{cj}}$) calculated by the combination weighting method.

Main Target	Facet for the First Layer	Facet Weight (w1ci)	Criterion (indicator) for the Second Layer	Dimension	Indicator Weight (w2cj)	Total Weight (Entropy-AHP wcj =w1ci×w2cj)
Suitable supplier selection	Product satisfaction (A)	0.5042	A1.Rate of qualified products	positive	0.4790	0.2415
			A2.Product price (thousand dollars)	negative	0.2985	0.1505
			A3.Rate of Product market share	positive	0.2225	0.1122
			Subtotal		1	---
	Supply innovation capability (B)	0.2122	B1.Supply capacity (kg/time)	positive	0.4951	0.1051
			B2.New product development rate (%)	positive	0.5049	0.1071
			Subtotal		1	---
	Service level ©	0.2836	C1. Delivery time (days)	negative	0.3152	0.0894
			C2. Delivery on time ratio (%)	positive	0.6848	0.1942
			Subtotal		1	---

**Table 7:** Euclidean distance measures from the positive-ideal solution (PIS).

Alternatives	$A_{1}$	$A_{2}$	$A_{3}$	$A_{4}$	$A_{5}$
$S^{+}$	0.0069	0.0149	0.0071	0.0101	0.0138

**Table 8:** Euclidean distance measures from the negative-ideal solution (NIS).

Alternatives	$A_{1}$	$A_{2}$	$A_{3}$	$A_{4}$	$A_{5}$
$S^{-}$	0.0123	0.0045	0.0105	0.0116	0.0061

**Table 9:** Relative proximity of the alternatives.

Alternatives	$A_{1}$	$A_{2}$	$A_{3}$	$A_{4}$	$A_{5}$
$\varphi_{i}$	0.6395	0.2326	0.5946	0.5350	0.3074

**Table 10:** The ranking of the options.

Options	$A_{1}$	$A_{2}$	$A_{3}$	$A_{4}$	$A_{5}$
**Rank**	1	5	2	3	4

**Table 11:** Facet weights of AHP-based technique for order preference by similarity to an ideal solution (TOPSIS) and entropy-AHP TOPSIS.

MCDM Method	Product Satisfaction (A)	Supply Innovation Capability (B)	Service Level (C)
AHP-based TOPSIS	0.3916	0.2815	0.3269
Entropy-AHP TOPSIS	0.5042	0.2122	0.2836

**Table 12:** Indicator weights of AHP-based TOPSIS and entropy-AHP TOPSIS.

MCDM Method	Rate of Qualified Products (A1)	Product Price (Thousand Dollars) (A2)	Rate of Product Market Share (A3)	Supply Capacity (kg/ time) (B1)	New Product Development Rate (%) (B2)	Delivery Time (days) (C1)	Delivery on Time Ratio (%) (C2)
AHP-based TOPSIS	0.4125	0.3759	0.2116	0.5293	0.4707	0.3917	0.6083
Entropy-AHP TOPSIS	0.4790	0.2985	0.2225	0.4951	0.5049	0.3152	0.6848

**Table 13:** Sensitivity analysis of the facet A weight (w1h1inw1c1) to the outcome of the alternatives. in entropy-AHP TOPSIS.

	w1h1= −50%	w1h1= −40%	w1h1= −30%	w1h1=−20%	w1h1= −10%	w1h1= 0	w1h1=10%	w1h1= 20%	w1h1= 30%	w1h1= 40%	w1h1= 50%
$A_{1}$	0.6406	0.6402	0.6400	0.6398	0.6396	0.6395	0.6394	0.6393	0.6392	0.6392	0.6406
$A_{2}$	0.2423	0.2391	0.2367	0.2350	0.2336	0.2326	0.2317	0.2310	0.2304	0.2299	0.2423
$A_{3}$	0.6000	0.5982	0.5969	0.5959	0.5952	0.5946	0.5941	0.5937	0.5934	0.5931	0.6000
$A_{4}$	0.5247	0.5283	0.5308	0.5326	0.5339	0.5350	0.5359	0.5366	0.5371	0.5376	0.5247
$A_{5}$	0.3664	0.3483	0.3343	0.3234	0.3146	0.3074	0.3014	0.2964	0.2921	0.2884	0.3664
